# Integrative Analysis of Gene Expression Data Including an Assessment of Pathway Enrichment for Predicting Prostate Cancer

**Published:** 2007-02-21

**Authors:** Pingzhao Hu, Celia M.T. Greenwood, Joseph Beyene

**Affiliations:** 1 Program in Genetics and Genomic Biology, The Hospital for Sick Children Research Institute, 15–706 TMDT, 101 College Street, Toronto, ON, M5G 1L7, Canada; 2 Department of Public Health Sciences, University of Toronto, Health Sciences Building, 155 College St, Toronto, ON, M5T 3M7, Canada; 3 Child Health Evaluative Sciences, The Hospital for Sick Children Research Institute, 555 University Ave, Toronto, ON, M5G 1X8, Canada

**Keywords:** pathway enrichment analysis, meta-analysis, data synthesis, probe-level test, prostate cancer, random effect models

## Abstract

**Background:**

Microarray technology has been previously used to identify genes that are differentially expressed between tumour and normal samples in a single study, as well as in syntheses involving multiple studies. When integrating results from several Affymetrix microarray datasets, previous studies summarized probeset-level data, which may potentially lead to a loss of information available at the probe-level. In this paper, we present an approach for integrating results across studies while taking probe-level data into account. Additionally, we follow a new direction in the analysis of microarray expression data, namely to focus on the variation of expression phenotypes in predefined gene sets, such as pathways. This targeted approach can be helpful for revealing information that is not easily visible from the changes in the individual genes.

**Results:**

We used a recently developed method to integrate Affymetrix expression data across studies. The idea is based on a probe-level based test statistic developed for testing for differentially expressed genes in individual studies. We incorporated this test statistic into a classic random-effects model for integrating data across studies. Subsequently, we used a gene set enrichment test to evaluate the significance of enriched biological pathways in the differentially expressed genes identified from the integrative analysis. We compared statistical and biological significance of the prognostic gene expression signatures and pathways identified in the probe-level model (PLM) with those in the probeset-level model (PSLM). Our integrative analysis of Affymetrix microarray data from 110 prostate cancer samples obtained from three studies reveals thousands of genes significantly correlated with tumour cell differentiation. The bioinformatics analysis, mapping these genes to the publicly available KEGG database, reveals evidence that tumour cell differentiation is significantly associated with many biological pathways. In particular, we observed that by integrating information from the insulin signalling pathway into our prediction model, we achieved better prediction of prostate cancer.

**Conclusions:**

Our data integration methodology provides an efficient way to identify biologically sound and statistically significant pathways from gene expression data. The significant gene expression phenotypes identified in our study have the potential to characterize complex genetic alterations in prostate cancer.

## 1. Introduction

Many statistical methods have been developed and applied to the molecular classification of cancers using gene expression profiling data. A popular paradigm for this kind of analysis is that a set of differentially expressed prognostic genes are first selected using a univariate method, such as the t-test, then a classifier is built on the selected genes (Golub et al. 1999; Dudoit et al. 2002; Ramaswamy et al. 2003; Tan et al. 2005b). There are some limitations to these methods: (1) they are for the most part developed purely based on computational or algorithmic grounds without using prior biological knowledge, such as pathway information, which is richly accumulated in the medical literatures and relevant public databases (Wei and Li, 2006); (2) it is hard to interpret individual genes on a list with many significant genes. Moreover, when several studies address the same question, these lists may identify very different sets of genes. For example, Sorlie et al. (2001), van’t Veer et al. (2002) and Ramaswamy et al. (2003) made attempts to predict survival of breast cancer patients, but the sets of survival-related genes identified in these studies had only a few genes in common. There are only 17 genes shared between the list of 456 genes from Sorlie et al. and the list of 231 genes from van’t Veer et al. and only 2 genes appeared in common between Sorlie et al. and Ramaswamy et al. Ein-Dor et al. (2005) reanalyzed the van t’Veer dataset in an attempt to explain the inconsistencies between lists from different studies. They found that the predictive power of several lists of survival-related genes, generated from the same data set, is similar and quite good, although the relative rankings of genes in different lists, on the basis of correlation with survival, change greatly. Moreover, membership in these prognostic lists is not necessarily indicative of the gene’s importance in cancer pathology. Since cancer is ‘caused’ or influenced by multiple gene variations more often than by a single gene, it is more reasonable to focus on pathways than on individual genes (Vogelstein and Kinzler, 2004). Therefore, recent focus has been on methods useful for discovering significant biological pathways which contribute to cancer. One of these innovative approaches is gene set enrichment analysis (GSEA) that focuses on evaluating gene expression data at the level of gene sets (Mootha et al. 2003; Subramanian et al. 2005). Starting with predefined gene sets belonging to particular pathways or sharing the same gene function categories, the GSEA method evaluates whether the elements of a given gene set tend to occur toward the top (or bottom) of a ranked gene list, according to their differential expression between two classes (such as normal and cancer samples) measured by signal to noise ratios (SNR) (Golub et al. 1999) or similar metric.

Another challenge of microarray data analysis is that although individual microarray studies can be highly informative in identifying individual genes (e.g. van’t Veer et al.’s 2002) or significant biological pathways (e.g. Mootha et al. 2003), it is still difficult to make a direct comparison among the results obtained by different groups addressing the same biological problem, since laboratory protocols, microarray platforms and analysis techniques used in each study may not be identical. Moreover, most individual studies have relatively small sample sizes, and hence prediction models trained in individual studies by using cross-validation are prone to over-fitting, leading to prediction accuracies that may be less robust and lack generalizability (Cruz and Wishart, 2006). Recent studies show that systematic integration of gene expression data from different sources can increase statistical power to detecting differentially expressed genes while allowing for an assessment of heterogeneity (Rhodes et al. 2002; Choi et al. 2003; Hu et al. 2005; Stevens and Doerge, 2005), and may lead to more robust, reproducible and accurate predictions.

In general, the approaches used for integration across studies fall into three broad categories. In the first approach, each data set is normalized and standardized and then the datasets are directly combined to appear to be a single experiment (Wang et al. 2006). This method is simple and can sometimes work well, but it cannot capture or appropriately cope with any inter-laboratory differences, which can be quite substantial even within the same technology (Irizarry et al. 2005). Variation in patient populations, environments, or lab conditions means that two studies may have different gene expression patterns, and a combined analysis ignores this source of variability. The second approach combines p-values from individual studies to estimate an overall p-value for each gene across all studies (Rhodes et al. 2002). Since in this case the method chosen to combine results across studies is based on the statistical confidence measure (the p-value), not on the expression levels, this strategy avoids direct comparisons of data, and hence avoids issues related to cross-platform differences in measurement or normalization. However, Hu et al. (2006a) showed that combining only p-values, while useful in obtaining more precise estimates of significance, may not indicate the direction of significance (e.g. up-or down-regulation). Moreover, a significant result from a large combined sample, based on the Fisher test combining p-values, does not necessarily correspond to a biologically important effect size (Rhodes et al. 2002). The third approach is based on integrating microarray expression values using random effect or fixed effect hierarchical models (Choi et al. 2003; Hu et al. 2005), in which the effect size estimate of a gene is used to measure the magnitude of treatment effect in a given study. Choi et al. (2003) demonstrated that their random effects model can lead to the discovery of small but consistent expression changes with increased sensitivity and reliability. The advantage of a random effects model is that variability between studies is estimated and taken into account.

Previous applications of random effects or fixed effects models to integrate the results of experiments performed using the Affymetrix technology have mainly focused on summarized probeset-level gene expression data (e.g. Hu et al. 2005). A probeset consists of 11–20 probe pairs where a probe is a short sequence of nucleotides in the coding region of the gene; the summary is a single representative measure of probeset expression. However, there may be additional information at the probe level that is lost by combining the probeset results into a single number (Bolstad, 2004; Elo et al. 2005). Some recent studies showed that methods based on probe-level models have much higher power to detect differentially expressed genes than summarized probeset-level approaches, in either individual studies (Bolstad, 2004), or across studies (Elo et al. 2005). For example, Bolstad (2004) developed a probe-level based test statistic for detecting differentially expressed genes in an individual study, in which parameters are estimated that account for variability across arrays and across probes. In the same vein, Elo et al. (2005) first calculated effect size for each probe in a given probeset, then obtained a summary (probeset-level) estimate of effect size estimate by averaging the probe-level estimates over the probes within each probe set.

The objectives of this study are twofold: The first is to demonstrate how to incorporate Bolstad’s probe-level -based test statistics into a random effects model (Choi et al. 2003; Hu et al. 2005) in order to integrate prostate cancer microarray expression data across studies; The second is to identify significant biological pathways from the integrative analysis and evaluate the power of the identified pathways for predicting prostate cancer. We compare the advantages and disadvantages of the probe-level based model with the traditional probeset-level based model from these two points of view.

## 2. Data sets and pre-processing

Data on gene expression in prostate tumours and controls were obtained from Welsh et al. (2001), Singh et al. (2002), LaTulippe et al. (2002), and Stuart et al. (2004). The datasets will be referred to by the name of the first author. All these datasets are either publicly available or obtainable upon request. Information about these datasets, such as the microarray platform, the number of samples available, and the data sources, is listed in Table 1. Using leave-one-out cross-validation (LOOCV), excellent predictive accuracy has been obtained for the Singh data based on both the K-nearest neighbour (KNN) model (Singh et al. 2002) and the top-scoring pair (TSP) algorithm (Tan et al. 2005a and Xu et al. 2005). In order to compare our predictive performance with those results, we randomly divided the Singh data into a training set and a testing set; each of these two datasets includes 25 normal samples and 26 cancer samples. Therefore, the Welsh data, the LaTulippe data, and the Singh training data were used to develop our predictive models (the “training data”), and the remaining data sets were used for testing the models (the “testing” data). First, the training data were used to identify differentially expressed genes and significant biological pathways for building models to predict primary prostate cancer. The predictive power of the selected genes and pathways were then evaluated using the testing data.

Since the Affymetrix microarray data sets in this meta-analysis were analyzed in two ways (at the probe-level and at the probeset-level), we normalized the probe-level perfect match (PM) and mismatch (MM) densities using the quantile normalization method (Bolstad et al. 2003) within each dataset. For the probeset-level model analysis, we then converted the quantile-normalized probe level data to a single expression measure for each probe set and each dataset, using the robust multi-array average (RMA) algorithm (Irizarry et al. 2003).

## 3. Methods

### 3.1 Modelling effect sizes to integrate gene expression patterns across studies

We used a random effect model of effect size measures to integrate gene expression patterns across studies (Choi et al. 2003; Hu et al. 2005). There are different ways to measuring effect size *y_g_* for gene *g* in any individual study. Here we present two methods: One is based on summarized probeset-level data (Choi et al. 2003; Hu et al. 2005); another is the one we recently developed (Hu et al. 2006b), which is based on the Affyme-trix probe-level data. In order to simplify the discussion, we only consider a comparison of two

Let *n_t_*, *n_c_* and *n* = *n_t_* + *n_c_* denote the number of treatment, control and total samples in the study, respectively.

#### 3.1.1 Measuring effect size using probe-level Affymetrix microarray data

The probe-level based effect size measure is derived from a recently proposed probe-level based test statistic for detecting differentially expressed genes (Bolstad, 2004; Bolstad, 2005). A probe-level model can be defined as follows: For each dataset assume that there are *I* probes for each probeset and *n* arrays. A probe-level model can be fitted using

(1) $$pm_{ij}=mm_{ij}+\alpha_{i}+\beta_{j}+{ɛ}_{ij}$$

where *i = 1, …, I* and *j = 1, …, n*, *pm_ij_* and *mm_ij_* are the pre-processed (normalized) log_2_ of the perfect match and mismatch intensities, respectively, *α_i_* represent probe effects and *β_j_* are array effects (on the log_2_ expression scale). The error is assumed to have mean zero and *Var*(*ɛ_ij_*) = *σ*^2^. To make the model identifiable, the constraint ∑*^I^_i_*_=1_ α*_i_* = 0 is used. Let β̑ be the estimated array effects and ∑̑ be the portion of the estimated variance-covariance matrix related to *β* from fitting the probe-level model (1). Let *V* be a contrast vector where element *j* of *V* is $\frac{1}{n_{c}}$ if array *j* is in group *c*, or $\frac{-1}{n_{t}}$ if array j is in group *t*. Then, a probe-level based **t**-test statistic (*t_pl_*) can be defined as

(2) $$t_{pl}=\frac{V^{\prime }\overset{̑}{\beta }}{\sqrt{V^{\prime }diag(\overset{̑}{\Sigma })V}}$$

Here *diag*(∑̑) means that the off-diagonal elements of ∑̑ are zero. This test statistic can be used to detect differential expression between the control group (*c*) and the treatment group (*t*). For each study, we define an effect measure for gene *g* by transforming the probe-level based t-statistic in (2) as follows:

(3) $$y_{g}=t_{pl}\sqrt{\frac{n_{t}+n_{c}}{n_{t}*n_{c}}}$$

The variance of this effect measure, *s*^2^*_g_*, can be estimated by

(4) $$\begin{array}{l} {\hat{s}}_{g}^{2}=\text{var}\left(t_{pl}\sqrt{\frac{n_{t}+n_{c}}{n_{t}*n_{c}}}\right)=\frac{n_{t}+n_{c}}{n_{t}*n_{c}}\\ {}*\text{var}\left(\frac{V^{\prime }\hat{\beta }}{\sqrt{V^{\prime }diag(\hat{\Sigma })V}}\right)=\frac{1}{n_{t}}+\frac{1}{n_{c}} \end{array}$$

#### 3.1.2 Measuring effect size using summarized probeset-level Affymetrix microarray data

A corresponding effect size for summarized probeset-level Affymetrix microarray data can be defined as

(5) $$y_{g}=\frac{\left({\bar{x}}_{gt}-{\bar{x}}_{gc}\right)}{s_{g}^{pool}}$$

where *x̄_gt_* and *x̄_gc_* are the sample means of gene expression values for gene *g* in treatment group *t* and control group *c* of a given study*,* respectively, and where *s^pool^_g_* is the pooled standard deviation (Hu et al. 2005). For a study with *n* samples, an approximately unbiased estimation of *y̑_g_* is given by *y̑_g_* = *y_g_* − 3* *y_g_* /(4*n* − 9) and its variance *s*^2^*_g_* can be estimated by

(6) $${\hat{s}}_{g}^{2}=\left(\frac{1}{n_{t}}+\frac{1}{n_{c}}\right)+\frac{{\hat{y}}_{g}^{2}}{2(n_{t}+n_{c})}$$

This definition of effect size is widely adopted in the meta-analysis literature (Hedges and Olkin 1985).

#### 3.1.3 Integrating effect sizes across studies

For each gene *g*, we have estimated its effect size *y_gm_*(*m = 1, …, M*) in *M* studies using equation (3) for probe-level analysis and using equation (5) for probeset-level analysis. A detailed description of the modelling techniques for integrating micro-array data across studies can be found in Hu et al. 2005. Let *μ_g_* denote the overall mean effect size of gene *g* in all *M* studies and *s*^2^*_gm_* be the effect size variance of gene g, measuring the sampling error for the *m^th^* study. Using a random effects model (Choi et al. 2003; Hu et al. 2005), the meta-analysis estimate for *μ_g_* can be calculated as:

(7) $${\hat{\mu }}_{g}=\frac{\Sigma_{m=1}^{M}w_{m}y_{gm}}{\Sigma_{m=1}^{M}w_{m}}$$

where the weights are given by *w_m_* = (*s*^2^*_gm_* + τ^2^)^−1^ and τ^2^ is the between-study variability (Choi et al. 2003). The variance of this estimator is obtained by

(8) $$Var({\hat{\mu }}_{g})=\frac{1}{\Sigma_{m=1}^{M}w_{m}}$$

A test statistic to evaluate the treatment effect of gene *g* across all *m* studies can then be computed as

(9) $$z_{g}=\frac{{\hat{\mu }}_{g}}{\sqrt{\text{var}({\hat{\mu }}_{g})}}$$

We evaluated the statistical significance of gene *g* by calculating the p-value corresponding to the z statistic, then we estimated the false discovery rates (FDR) for each significance level, to take into account the number of tests performed (Benjamini and Hochberg, 1995). We refer the approach of estimating *z_g_* using the probe-level based test statistic as the Probe-Level Model (PLM) and we refer to the method based on the probeset-level test statistic as the ProbeSet-Level Model (PSLM).

### 3.2 Pathway-based learning models for predicting prostate cancer

#### 3.2.1 Selecting gene sets

Pathway-based models assume that members in a set of genes are known to belong to the same pathway or have the same function. Such prior biological knowledge can be derived from many public sources, such as Gene Ontology (GO) (Ashburner et al. 2000) or the Kyoto Encyclopedia of Genes and Genomes (KEGG) (Kanehisa and Goto, 2002). The former is a database of controlled vocabulary gene annotations describing the biological processes, molecular functions and cellular localizations of genes, while the latter is a pathway resource, which contains graphical representations of cellular processes. Since pathways involving multiple processes and functions are not well represented in GO, we defined the gene sets used in this study based on KEGG.

#### 3.2.2 Mapping differentially expressed probes (genes) to predefined gene sets

Using the probe identifiers in the Affymetrix annotation table for HGu95Av2 GeneChips (obtained from http://www.affymetrix.com/support/technical), we mapped the selected differentially expressed probes (FDR-adjusted p-values< = 0.05) to KEGG pathways via LocusLink identifiers using the function ‘*probes2Path’* in the Category package of bioconductor (www.bioconductor.org). It should be noted that some LocusLink identifiers may not be mapped to any known pathways in KEGG due to the limited number of pathways in the database.

#### 3.2.3 Evaluating significance for mapped gene sets

There are different ways to test for an excess of differentially expressed genes in the same pathway. We used the “gene set enrichment test” implemented in the limma R package (Smyth, 2004). The approach uses the Wilcoxon signed rank test to compute a p-value to test the hypothesis that a given mapped gene set tends to be more highly ranked than would be expected by chance. The ranking must be based on a t-like test statistic, and here we used the z statistics for PLM and PSLM described in Section 3.1. The test is essentially a streamlined version of the GSEA approach introduced by Mootha et al. (2003).

#### 3.2.4 Pathway-based learning models using support vector machines (SVMs)

For each significant biological pathway identified from the analysis in Section 3.2.3, we built two simple linear kernel function-based SVM classification models, for PLM and PSLM, respectively, using the training data and evaluated their performance using the testing data (see Table 1). A detailed description of the mathematics behind SVM can be found in (Vapnik, 1998). In this study, we used the SVM algorithm implemented in the e1071 package in the R Project for Statistical Computing (http://www.r-project.org/). The performance of the pathway-based SVM model was evaluated based on prediction accuracy, namely, the proportion of correctly predicted samples out of all samples in a given testing set.

## 4. Results

### 4.1 Genes showing significant expression patterns with tumour differentiation

**Figure 1:**
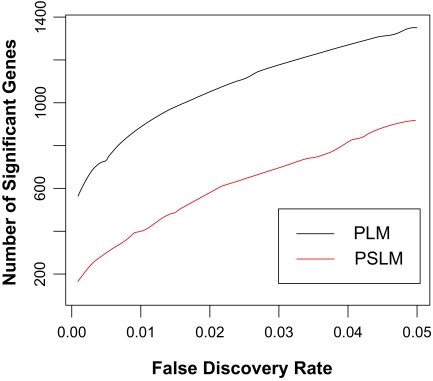
Number of differentially expressed genes as a function of false discovery rate (FDR) thresholds.

We identified 12,600 common probesets across the three training sets as shown in Table 1, and PLM and PSLM were applied to the common probesets. Figure 1 shows the number of differentially expressed genes identified by integrative analysis of the common expression patterns in the three training sets for different thresholds of FDR-adjusted p-values. It can be seen that a large number of differentially expressed genes were obtained using these two models. For example, by setting a threshold of FDR = 0.05, we obtained 1350 differentially expressed genes using PLM and 917 differentially expressed genes using PSLM. In general, the PLM method identified more differentially expressed genes than the PSLM approach for FDR thresholds between 0.00 and 0.05 (Fig. 1). Moreover, the absolute values of the z statistics (a measure of significance) for genes identified by PLM are much larger than those identified by PSLM. The sets of significantly expressed genes, for FDR-adjusted p-values less than 0.05, are provided in supplementary Tables 1 and 2 for PLM and PSLM, respectively. There are 672 shared genes in these two lists. A close examination of the top 50 gene expression signatures in the gene lists of supplementary Tables 1 and 2 indicates more previously known functionally important genes at the top of the PLM list. For example, HPN, which was identified only by PLM, is functionally linked to the hepatocyte growth factor/MET pathway and has been found to be highly expressed in prostate tumours (Singh et al. 2002). This gene was also included in the 16-gene K-nearest neighbour (KNN) model of Singh et al. (2002), and in the TSP model of Xu et al. (2005). Another gene on the top of PLM list, FASN, is a known tumour marker; Welsh et al. (2001) found strong and specific immunopositivity in malignant epithelium in all of 10 cancer patients when they stained tissue sections with a monoclonal antibody against FASN.

**Figure 2:**
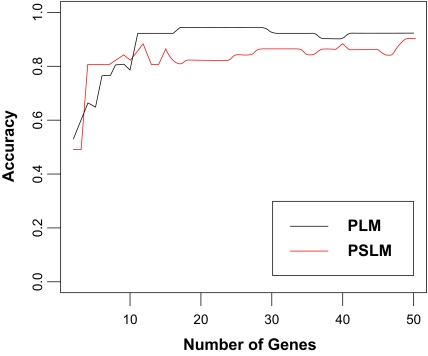

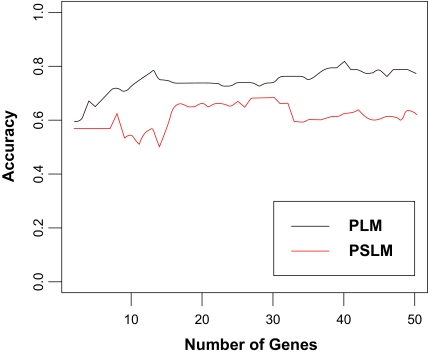
Predictive accuracy of the SVM models, as a function of the number of differentially expressed genes used for prediction: (a) Singh testing data; (b) Stuart testing data.

We evaluated the discriminative power of the differentially expressed genes in Supplementary Tables 1 and 2 using linear kernel function-based SVM models built on the training datasets, and varying the number of predictors between 1 and all selected genes, and the predictive accuracy stopped improving after 50 genes. The performance of these predictors was tested separately on the testing data portion of Singh et al. (2002)’s data, and on an independent dataset (Stuart et al. 2004). The classification accuracies are presented in Figures 2(a) for the Singh data, and 2(b) for the Stuart data. Models were built and tested separately for each number of genes included as predictors. Genes obtained from the meta-analyses of the training sets were ranked by the adjusted p-values for inclusion in the prediction models, so that, for example, the models containing 10 genes used the 10 genes with the smallest adjusted p-values.

For the Singh data, genes identified by PLM usually have better prediction accuracies than those identified by PSLM. The best prediction accuracy is 94.1% using 17 genes identified by PLM and 90.2% using 49 genes selected by PSLM. For the Stuart data, PLM also outperforms PSLM. Our best accuracies for the Stuart data are 81.8% for PLM model using 40 genes and 68.2% using 27 genes for PSLM.

### 4.2 Significantly enriched KEGG pathways for differentially expressed genes with tumour differentiation

We tested both sets of significantly expressed genes (shown in Supplementary Tables 1 and 2) for identifying significantly enriched KEGG pathways. We identified 129 and 116 pathways showing evidence for enrichment with p-values less than 0.01, using PLM and PSLM, respectively. There are 113 shared pathways in these two sets of pathways. Tables 2 and 3 show the top 20 significantly enriched pathways identified in each of these two lists. These top pathways are all highly significantly enriched by both methods of analysis. Among the top 20 pathways, 15 are in common across the two methods for integrative analysis.

Prediction models using SVM were developed using the pathway-identified sets, and strong predictive power can be seen in Tables 2 and 3 for many of the pathways and for both integrative methods. However, the predictive power appears better for models built on the PLM integrative analysis than the PSLM analysis. For example, we found the insulin signalling pathway has consistently strong predictive power in the two test sets using either PLM or PSLM. Using PLM, this pathway, including 28 genes, has prediction accuracy of 92.2% for the Singh data and 79.5% for the Stuart data. In PSLM, however, this pathway now represented by only 21 genes, has only 80.4% and 73.9% classification accuracy for the Singh data and the Stuart data, respectively.

## 5. Discussion

In this study, we used a recently developed method to integrate Affymetrix expression data across studies (Hu et al. 2006b). The idea is based on a probe-level based test statistic developed for testing differentially expressed genes in individual studies (Bolstad, 2004; Bolstad, 2005). We incorporated this test statistic into a classic random-effects model for integrating data across studies. When this new method was compared with a more traditional method to summarize probeset-level test statistics across different studies (Choi et al. 2003), the sets of genes and pathways identified by PLM were more statistically significant and biologically sound. The PLM identified more differentially expressed genes and pathways than the PSLM. Moreover, the PLM identified some biologically validated genes contributing to prostate cancer, which have not been detected by the PSLM. Using SVM-based classifiers, the genes and pathways identified by PLM have better predictive power in most cases than those identified by PSLM.

Our models show competitive predictive capability when compared to the previous analyses of these data. For example, Singh et al. (2002) selected 1–256 genes by using the signal-to-noise statistic (Golub et al. 1999) and measured differential expression between normal and tumour prostate samples. For each of the 256 sets of genes, they built a KNN classifier and estimated prediction accuracy using leave-one-out cross-validation (LOOCV). The range of the prediction accuracy was 86%–92%, corresponding to between 4–256 genes. Tan et al. (2005a) showed the predictive accuracy for 7 classifiers (TSP, K-TSP, C4.5 decision tree (DT), Naïve Bayes (NB), KNN, SVM and prediction analysis of microarrays (PAM)) based on LOOCV for the Stuart dataset. Their best accuracies were 67.6%, 75.0%, 64.8%, 73.9%, 69.3%, 76.1% and 79.6%, respectively. Some studies show that LOOCV overestimates accuracy relative to accuracies based on 10 fold cross-validation (Ambroise and McLachlan, 2002). However, other studies also pointed out that classification accuracy, when determined by cross-validation using the same data set from which the class predictor was derived, may be overestimated (see supplementary materials in Lapointe et al. 2004).

Traditional microarray-based cancer prediction approaches use only differentially expressed genes as biomarkers to discriminate classes of cancer and normal samples. However, a large proportion of such genes are irrelevant and functional correlations among those genes are ignored. Since the genes with the best discriminative power are likely to correspond to a limited set of biological functions or pathways, it is rational to focus on these key functional expression patterns/pathways for cancer prediction. This approach may then provide clues as to the types of biological processes that underlie the expression patterns of sets of genes. We found several pathways defined in the KEGG database could accurately discriminate prostate cancer samples from control samples. Although the best performance of the pathway-based prediction models (e.g. 92.2% prediction accuracy was obtained for Singh testing data using 28 genes in insulin signalling pathway identified by PLM) is slightly worse than the best prediction accuracy based solely on the top genes (e.g. 94.1% prediction accuracy was obtained for the same data using 17 the most significantly expressed genes), the set of significantly enriched pathways showing the strongest correlation to prostate tumour expression patterns is likely to be of greater biological interest. Such pathways might indicate one or more processes as acting drivers related to prostate cancer. Furthermore, this enables a large set of genes or probesets, identified by the integrative analysis testing for differential expression, to be considered in the subsequent pathway analysis and predictive modelling. Hence this pathway analysis approach may reduce the concern over the variability in the specific genes given in the top ranking by any one dataset or any one analysis. There appears to be more consistency in the represented pathways across the two integrative methods than in the top genes. It should be noted that there are also other carefully curated pathway sets (Subramanian et al. 2005) and gene sets, such as gene ontology (Harris et al. 2004), that could be used for this purpose. We did not explore them, since our focus was on comparing the performance of PLM and PSLM rather than on identifying the best pathways for predicting prostate cancer. In this study, we just focused on the prediction power of individual pathways. It will be also interesting to evaluate the interactions among pathways and their effects on cancer prediction.

In this paper, we have used a probe-level based statistic (Bolstad, 2004; Bolstad, 2005) to define an effect size for the purpose of data integration. The test statistic treats probes as replicates which might lead to a biased variance estimate for the t statistic, which in turn will have impact on the effect size defined at the probe-level. This issue warrants further investigation in the future. For example, one may redefine the effect size using a correction factor similar to the factor proposed by Hedges and Olkin (1985) in the case of the standardized mean difference effect measure.

## Figures and Tables

**Table 1:** Training and test data sets.

Data Set	Platform	Number of Probe Sets/Spots	Number of Normal Samples	Number of Cancer Samples	Reference	Source of Raw Data
Training Sets	Affymetrix (HG_U95Av2)	12600	25	26	Singh et al. (2002)[1]	Supplement
	Affymetrix (HG_U95Av2)	12626	8	25	Welsh et al. (2001)[2]	Author
	Affymetrix (HG_U95Av2)	12626	3	23	LaTulippe et al. (2002)	GEDP[3]
Testing Sets	Affymetrix (HG_U95Av2)	12600	25	26	Singh et al. (2002)[1]	Supplement
	Affymetrix (HG_U95Av2)	12625	50	38	Stuart et al. (2004)	GEO[4]

1 The Singh data set was randomly divided into a training set (51 arrays) and a testing set (51 arrays)

2 The numbers of normal and cancer samples shown in original papers are 9 and 24 respectively. The author suggested that we treat the data as 8 normal samples and 25 cancer samples when they sent us their raw data (CEL files)

3 The Gene Expression Data Portal (GEDP), National Cancer Institute

4 GEO: Gene Expression Omnibus groups: treatment (*t*) and control (*c*) groups in a study.

**Table 2:** The top 20 significantly enriched pathways for the set of significantly differentially-expressed genes identified using PLM (Supplementary Table 1), together with their predictive accuracies in the test datasets.

Pathway ID	p-value	# of Genes*	Accuracy of Singh testing data	Accuracy of Stuart testing data	Pathway Name
04810	0	33	0.824	0.761	Regulation of actin cytoskeleton
04910	0	28	0.922	0.795	Insulin signaling pathway
00230	0	29	0.686	0.591	Purine metabolism
04010	0	46	0.745	0.818	MAPK signaling pathway
04020	0	29	0.824	0.568	Calcium signaling pathway
04510	0	35	0.804	0.693	Focal adhesion
00190	5.55E-16	23	0.804	0.67	Oxidative phosphorylation
04514	8.88E-16	23	0.843	0.648	Cell adhesion molecules (CAMs)
00240	1.78E-14	21	0.745	0.557	Pyrimidine metabolism
04070	3.44E-14	20	0.765	0.705	Phosphatidylinositol signaling system
01430	1.25E-13	19	0.843	0.739	Cell Communication
04060	5.75E-13	18	0.765	0.682	Cytokine-cytokine receptor interaction
04530	2.93E-12	17	0.784	0.602	Tight junction
00330	4.09E-12	17	0.882	0.739	Arginine and proline metabolism
04310	1.01E-11	16	0.725	0.682	Wnt signaling pathway
00480	2.40E-11	16	0.843	0.67	Glutathione metabolism
04540	4.48E-11	15	0.686	0.818	Gap junction
04720	4.61E-11	15	0.765	0.773	Long-term potentiation
04670	4.96E-11	15	0.824	0.705	Leukocyte transendothelial migration
04512	5.93E-11	15	0.745	0.795	ECM-receptor interaction

* The number of genes used in building models for prostate cancer prediction.

**Table 3:** The top 20 significantly enriched pathways found for the set of significantly differentially-expressed genes identified using PSLM (Supplementary Table 2), together with the predictive accuracies in the test datasets.

Pathway ID	p-value	# of Genes	Accuracy in the Singh testing data	Accuracy in the Stuart testing data	Pathway Name
03010	0	32	0.784	0.693	Ribosome
04010	3.33E-16	26	0.784	0.455	MAPK signaling pathway
04810	5.66E-14	22	0.745	0.602	Regulation of actin cytoskeleton
00230	6.13E-14	21	0.686	0.591	Purine metabolism
04910	1.41E-13	21	0.804	0.739	Insulin signaling pathway
04514	3.08E-13	20	0.824	0.614	Cell adhesion molecules (CAMs)
04020	4.49E-13	20	0.784	0.602	Calcium signaling pathway
04510	1.22E-12	19	0.765	0.58	Focal adhesion
00190	1.31E-10	16	0.843	0.693	Oxidative phosphorylation
04664	1.17E-09	14	0.686	0.5	Fc epsilon RI signaling pathway
04540	1.17E-09	13	0.706	0.5	Gap junction
04060	3.16E-09	13	0.725	0.591	Cytokine-cytokine receptor interaction
00240	3.94E-09	13	0.843	0.705	Pyrimidine metabolism
00480	4.95E-09	13	0.784	0.591	Glutathione metabolism
04520	5.33E-09	13	0.784	0.466	Adherens junction
04070	4.75E-08	11	0.686	0.739	Phosphatidylinositol signaling system
04080	7.64E-08	11	0.706	0.614	Neuroactive ligand-receptor interaction
04670	7.75E-08	11	0.784	0.602	Leukocyte transendothelial migration
04512	1.33E-07	10	0.765	0.693	ECM-receptor interaction
04360	1.58E-07	10	0.745	0.523	Axon guidance
